# Metabolic Syndrome Causing Cognitive Impairment in Patients With Schizophrenia: A Systematic Review

**DOI:** 10.7759/cureus.47587

**Published:** 2023-10-24

**Authors:** Ayushi Saxena, Dhara Patel, Ismat E Ayesha, Neetha R Monson, Nimra Klair, Utkarsh Patel, Safeera Khan

**Affiliations:** 1 Internal Medicine, California Institute of Behavioral Neurosciences and Psychology, Fairfield, USA

**Keywords:** cognitive neuroscience, diabetes obesity, schizophrenia, cognitive impairement, ms: metabolic syndrome

## Abstract

Schizophrenia often exhibits characteristics like cognitive deficits, and individuals with the condition are at a higher risk of developing metabolic syndrome. The effect of metabolic syndrome on schizophrenia-related cognitive impairment is still unknown, though. This systematic review aims to investigate the association between metabolic syndrome and cognitive impairment in patients with schizophrenia, specifically focusing on neurocognitive and social cognitive performance. Schizophrenia significantly strains the public healthcare system since it necessitates tremendous resources and care to support those suffering from the condition. Furthermore, patients with schizophrenia are more susceptible to developing obesity than the general population, leading to a higher possibility of developing metabolic syndrome. The gut microbiota has been recognized as a critical regulator of bidirectional interactions between the central nervous system and the gastrointestinal tract. Emerging evidence suggests that dysbiosis of the gut microbiota is closely linked to the development of both schizophrenia and obesity, sharing common pathophysiological mechanisms, particularly immune inflammation. In this systematic review, we examine the existing literature to explore the relationship between metabolic syndrome and cognitive impairment in individuals with schizophrenia. By synthesizing available evidence on neurocognitive and social cognitive performance, we aim to provide a comprehensive understanding of the association between metabolic syndrome and cognitive deficits in schizophrenia. The findings from this review will contribute to our knowledge of the complex interplay between metabolic abnormalities, gut microbiota dysbiosis, and cognitive impairments in patients with schizophrenia. This understanding may facilitate the development of novel interventions targeting metabolic syndrome as a potential avenue for improving cognitive outcomes in individuals with schizophrenia.

## Introduction and background

Metabolic syndrome is a complex condition characterized by a cluster of metabolic abnormalities that often occur together, increasing the risk of various health issues. The components of metabolic syndrome include high blood pressure, abdominal obesity, high blood sugar levels, high triglycerides, and low levels of high-density lipoprotein (HDL) cholesterol. This syndrome is linked with an increased risk of developing several other health conditions and diseases affecting various systems in the body. The American Heart Association (AHA) defines "Metabolic syndrome as a cluster of five interconnected conditions that heighten the risk of heart disease, diabetes, stroke, and various other health complications. The diagnosis of metabolic syndrome is established when an individual presents with three or more of these risk factors: elevated blood glucose levels, reduced levels of HDL ("good") cholesterol in the bloodstream, elevated triglyceride levels in the blood, a larger waist circumference indicative of an "apple-shaped" body, and high blood pressure [1]."

The American Psychiatric Association states that "Schizophrenia is a chronic brain disorder that affects less than one percent of the US population. In the active phase of schizophrenia, symptoms can include delusions, hallucinations, disorganized speech, trouble thinking, and lack of motivation [2]." According to the Diagnostic and Statistical Manual of Mental Disorders (5th ed.), "Schizophrenia is a complex mental disorder characterized by a range of symptoms that persist for at least six months, with a significant impact on an individual's daily life [3]. To diagnose schizophrenia, specific criteria must be met, including the presence of at least two of the following symptoms for a significant portion of a one-month period: delusions, hallucinations, disorganized speech, grossly disorganized or catatonic behavior, or negative symptoms [3]. Additionally, these symptoms must lead to a noticeable decline in functioning compared to the individual's previous level [3]. The diagnosis excludes schizoaffective disorder and mood disorders with psychotic features, and it cannot be attributed to substance abuse or other medical conditions [3]. In cases where there's a history of autism spectrum disorder or a childhood communication disorder, a diagnosis of schizophrenia is made only if prominent delusions or hallucinations are also present for a specific duration [3]."

Cognitive impairment is a wide phrase that refers to varying degrees of difficulties in cognitive activities such as thinking, memory, attention, reasoning, and problem-solving [2]. It can range from moderate cognitive impairments that have little influence on everyday life to severe impairments that interfere with a person's ability to conduct normal duties and preserve independence. Cognitive impairment can be caused by a variety of factors, such as neurological abnormalities, brain traumas, mental health issues, or age-related decreases in cognitive function [2,3]. Individuals with cognitive impairment might vary greatly in terms of severity and specific cognitive domains impacted.

Metabolic syndrome is markedly more prevalent among individuals with schizophrenia, impacting 40-60% of patients, a rate significantly higher than that seen in the general population, as evidenced by a long-term study involving individuals with schizophrenia and bipolar disorder using data from the Suffolk County Mental Health Project cohort, which included consecutive initial admissions with psychosis from September 1989 to December 1995 and a subsequent 20-year follow-up [4]. Schizophrenia patients who are obese often exhibit lower cognitive performance and physical fitness and have a shorter life expectancy than those with normal weight [5]. As a result, obesity is considered a significant factor contributing to unfavorable psychiatric outcomes in schizophrenia.

Individuals with schizophrenia are at greater risk for metabolic syndrome, associated with cognitive deficits in the general population. In schizophrenia, metabolic syndrome may significantly contribute to cognitive impairment [6]. A significant decline in life expectancy, mostly as a result of greater incidence of cardiovascular disease (CVD), is a hallmark of schizophrenia, which is a severe and crippling disorder [7]. One significant risk factor for CVD in individuals with schizophrenia is metabolic syndrome and its various diagnostic criteria [8]. Metabolic syndrome encompasses a range of abnormal clinical and metabolic findings that serve as predictors of CVD [8]. The Clinical Antipsychotic Trials of Intervention Effectiveness (CATIE) study found that patients with schizophrenia are far more likely to have metabolic syndrome than those in general. According to the National Cholesterol Education Program (NCEP) and American Heart Association (AHA) standards, the study found that the prevalence rates for metabolic syndrome were 40.9% and 42.7%, respectively. Compared to the prevalence in males, which was 36.0% and 36.6% (p=0.0002), it was significantly greater in females (51.6% and 54.2%).

**Figure 1:**
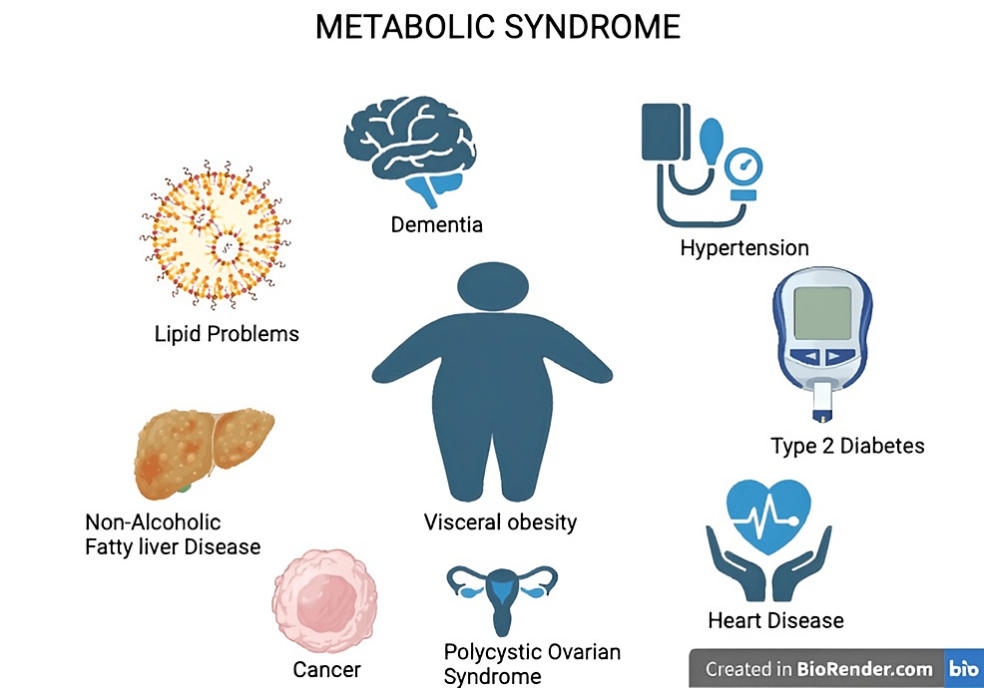
The key associations of metabolic syndrome with other systems Image credits: Ayushi Saxena Created in Biorender.com [12]

In comparison, the overall US population had a 23.7% age-adjusted prevalence of metabolic syndrome [9]. High rates of obesity, which may be influenced by the increased use of atypical antipsychotics, and lifestyle choices are contributing causes to this gap. Furthermore, diabetes, hypertension, and other cardiovascular risk factors may develop in people with schizophrenia who have metabolic syndrome. This susceptible population experiences higher morbidity and death due to these disorders, which are twice as common as in the general population, as evidenced by a study of 159 patients with schizophrenia or schizoaffective disorder. About 43.34% met the NCEP adult panel III criteria for metabolic syndrome, with patients without metabolic syndrome demonstrating significantly superior performance in processing speed, attention/vigilance, working memory, and problem-solving tests compared to those with metabolic syndrome, who exhibited lower cognitive domain scores; notably, greater waist circumference was associated with lower attention/vigilance scores, higher HDL levels were associated with higher attention/vigilance scores, and elevated triglyceride levels were associated with lower attention/vigilance scores, even after Bonferroni correction [10]. Evidence suggests that metabolic syndrome and its individual diagnostic criteria are risk factors for clinically significant cognitive impairment in both the general population and individuals with schizophrenia [10]. Schizophrenia is characterized by significant cognitive impairment, closely related to functional consequences. It has been seen to endure the duration of the illness and only slightly improve with antipsychotic therapy [10,11]. However, the specific impact of metabolic syndrome on cognitive functioning in individuals with schizophrenia remains unclear. Hence, the primary objective of this systematic review is to evaluate the available evidence regarding the association between metabolic syndrome and cognitive impairment in patients with schizophrenia. We aim to compare the association between metabolic syndrome patients and its contributing components and several neurocognitive and social domains [10]. Figure 1 explains metabolic syndrome and its key associations.

## Review

Methods

Search Strategy

This systematic review was conducted using the Preferred Reporting Items for Systematic Review and Meta-Analysis (PRISMA) 2020 guidelines. To identify relevant literature for our review, we conducted comprehensive searches on several databases, including PubMed, PubMed Central (PMC), PubMed (MESH), MDPI, JamaNetwork, Google Scholar, and ScienceDirect. We employed various combinations of keywords such as "schizophrenia and metabolic syndrome," "cognitive dysfunction and psychotic disorders," among others, to ensure a comprehensive search across all selected databases. Additionally, in PubMed, we implemented a specific search strategy alongside these keywords to further refine our search and identify pertinent literature in the field.

PubMed's MeSH database is as follows: ("Schizophrenia" [MeSH Terms] OR "Schizophrenia" [All Fields]) AND ("MetabolicSyndrome" [MeSH Terms] OR "Metabolic Syndrome X" [All Fields] OR "Metabolic Syndrome" [All Fields] OR "Insulin Resistance" [All Fields] OR "Obesity" [All Fields] OR "Dyslipidemias" [All Fields]) AND ("Cognition Disorders" [MeSH Terms] OR "CognitiveDysfunction" [All Fields] OR "Cognitive Impairment" [All Fields] OR "Neuropsychological Tests" [All Fields]. The outcomes of the search strategies are illustrated in Table 1.

**Table 1:** The databases used and the identified numbers of papers for each database MeSH: Medical Subject Headings

Search strategy	Database used	Number of papers identified
(Schizophrenia) AND (Diabetes) OR (Metabolic Syndrome) AND (Cognitive Impairment) OR (Neurocognitive Impairment)	MDPI	2807
("Schizophrenia"[MeSH Terms] OR "Schizophrenia"[All Fields]) AND ("Metabolic Syndrome"[MeSH Terms] OR "Metabolic Syndrome X"[All Fields] OR "Metabolic Syndrome"[All Fields] OR "Insulin Resistance"[All Fields] OR "Obesity"[All Fields] OR "Dyslipidemias"[All Fields]) AND ("Cognition Disorders"[MeSH Terms] OR "Cognitive Dysfunction"[All Fields] OR "Cognitive Impairment"[All Fields] OR "Neuropsychological Tests"[All Fields])	PubMed (MeSH)	127
(Schizophrenia) AND (Metabolic syndrome)) AND (cognitive impairment)	PubMed	66
("Metabolic Syndrome" OR "Insulin Resistance" OR "Dyslipidemia") AND ("Schizophrenia" OR "Psychotic Disorders") AND ("Cognitive Impairment" OR "Neurocognitive Disorders")	PubMed	42
(“Metabolic syndrome“) AND (“Cognitive Impairment”) AND (“Schizophrenia”)	ScienceDirect	694
(“Metabolic syndrome”) (“obesity“) (“cognitive impairment“) (“schizophrenia diabetes“)	JamaNetwork	2000
"Schizophrenia and metabolic syndrome," "Cognitive dysfunction and Psychotic Disorders,"	Google Scholar	86

Inclusion and Exclusion Criteria

Our study focused on the most recent literature published within the past decade. We specifically selected articles written in English or those with available full-text English translations. To ensure relevance to our research topic, we only included studies that involved human participants. Any articles where the full text could not be accessed were excluded from our review. We also excluded studies investigating metabolic syndrome or cognitive impairment in individuals without schizophrenia. We also did not include gray literature or proposal papers in our analysis. The exclusion and inclusion criteria are listed in Table 2.

**Table 2:** Exclusion and inclusion criteria DSM-5: Diagnostic and Statistical Manual of Mental Disorders 5, ICD: International Classification of Diseases

Exclusion criteria	Inclusion criteria
Grey literature	Papers written and published in the English language
Studies that primarily focus on pediatric or adolescent populations	Study participants: adults (18 years and above) diagnosed with schizophrenia according to established diagnostic criteria (DSM-5, ICD-10)
Studies without a clear assessment of metabolic syndrome or cognitive impairment	Metabolic syndrome: studies that assessed the presence of metabolic syndrome in a patient, considering establishing diagnostic criteria such as the National Cholesterol Education Program Adult Treatment Panel III (NCEP ATP III), International Diabetes Federation (IDF), or other widely accepted definition
Non-human studies	Cognitive impairment: studies that evaluate cognitive function using validated neuropsychological tests or standardized, cognitive assessments in patients with schizophrenia

Quality Assessment and Selection Process

The shortlisted articles were imported into EndNote (Clarivate, London, United Kingdom), and duplicate papers were eliminated. Subsequently, each article underwent screening based on titles and abstracts. The remaining articles were then subjected to a thorough evaluation of their full texts, and only those deemed relevant were included in the assessment. Inclusion and exclusion criteria were carefully applied, resulting in the final selection of articles that met the specified criteria.

The shortlisted articles underwent a rigorous quality assessment using appropriate quality appraisal tools. The Newcastle-Ottawa tool was employed to evaluate the quality of observational studies, while the Assessment of Multiple Systematic Reviews (AMSTAR) tool was utilized to assess the quality of systematic reviews. For narrative reviews, the Scale for the Quality Assessment of Narrative Review Articles (SANRA) was applied. Only studies that met the criteria of the quality appraisal were included in the systematic review, ensuring that only high-quality studies were considered for analysis. The quality appraisal for each study is shown in Table 3, using the appropriate evaluation technique for each type of study.

**Table 3:** Cochrane bias table for quality appraisal

Study ID	Randomization	Allocation concealment	Incomplete outcome data	Selective outcome reporting	Other biases
Wu et al. [5]	High	Unclear	Low	High	Low
Bora et al. [6]	Medium	Medium	Low	High	Low
Correll et al. [7]	High	High	Low	High	Low
Kern et al. [13]	High	High	Low	High	Low
Ojeda et al. [14]	Medium	High	Low	High	Low
Cryan et al. [15]	Unclear	Low	Low	Medium	Low
Li et al. [16]	Medium	High	Low	High	Low

The study characteristics and their findings are discussed below in Table 4.

**Table 4:** Study characteristics MATRICS: Measurement and Treatment Research to Improve Cognition in Schizophrenia, PANSS: Positive and Negative Syndrome Scale

Study	Study design	Population	Findings
Wu et al. [5]	Systematic review	Not specified	Gut microbial dysbiosis has been found in both schizophrenia and obesity, as have disruptions in the gut-brain axis, particularly gut-derived inflammation
Correll et al. [7]	Large-scale meta-analysis	3,211,768 patients and 113,383,368 controls	Increased risk of cognitive impairment in schizophrenia patients
Kern et al. [13]	Results from the MATRICS psychometric and standardization study	The study included 176 persons with schizophrenia or schizoaffective disorder and 300 community residents	Patients with schizophrenia have severe impairments in processing speed and working memory
Ojeda et al. [14]	Statistical analysis	One hundred patients with schizophrenia and 53 healthy adults	Schizophrenia is likely to have a core cognitive defect of slowed processing speed
Cryan et al. [15]	Abstract	Not specified	Unclear
Li et al. [16]	Cross-sectional study	Chinese population: female: 44%, male: 51%	Microbial diversity phylum: *Firmicutes* and *Actinobacteria* genus: *Adlercreutzia*, *Anaerostipes*, *Ruminococcus*, and *Faecalibacterium*. *Lactobacillus*, *Succinivibrio*, *Mogibacterium*, and *Corynebacterium*. PANSS and *Succinivibrio* scores are positive. PANSS and *Corynebacterium* scores are negative

Results

Study Identification and Selection

**Figure 2:**
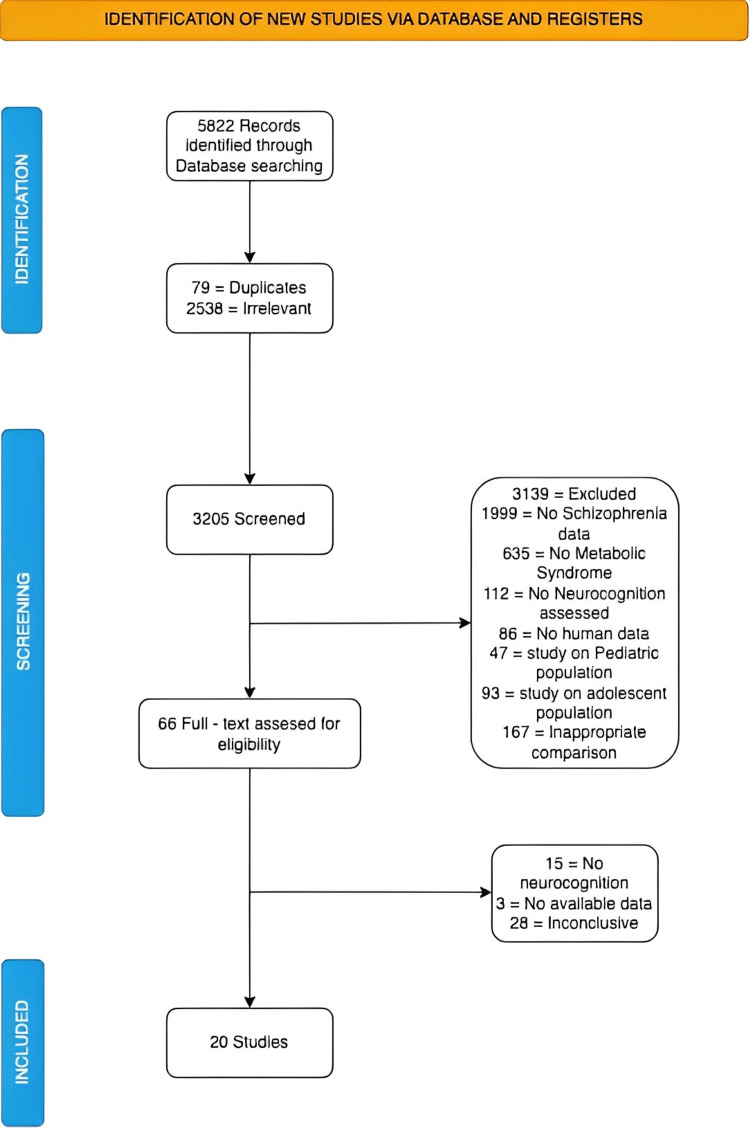
PRISMA flowchart PRISMA: Preferred Reporting Items for Systematic Reviews and Meta-Analyses [17]

A comprehensive search across all selected databases yielded a total of 5,822 articles. After removing duplicates (79 articles) and irrelevant studies (2,538 articles) through initial screening based on titles and abstracts, a total of 66 articles remained for further evaluation. The full-text articles from this selection were thoroughly assessed for eligibility and quality, resulting in the final inclusion of 20 articles for the review. The detailed selection process is presented in the PRISMA flowchart in Figure 2.

Discussion

The gut-brain axis, a pathway that transmits information in both directions between the gastrointestinal tract and the brain, is essential for connecting these two systems. The gut microbiota is known to be an important regulator of this axis, which is known as the microbiota-gut-brain axis. On one hand, the gut microbiota can influence brain development by altering neuronal, immunological, and endocrine pathways, regulating mood, cognition, and behavioral traits in the host. The brain, on the other hand, may control gastrointestinal function and maintain homeostasis via neuronal and endocrine pathways, thereby exerting an influence on the composition, structure, and functionality of the gut microbiota [5,15].

**Figure 3:**
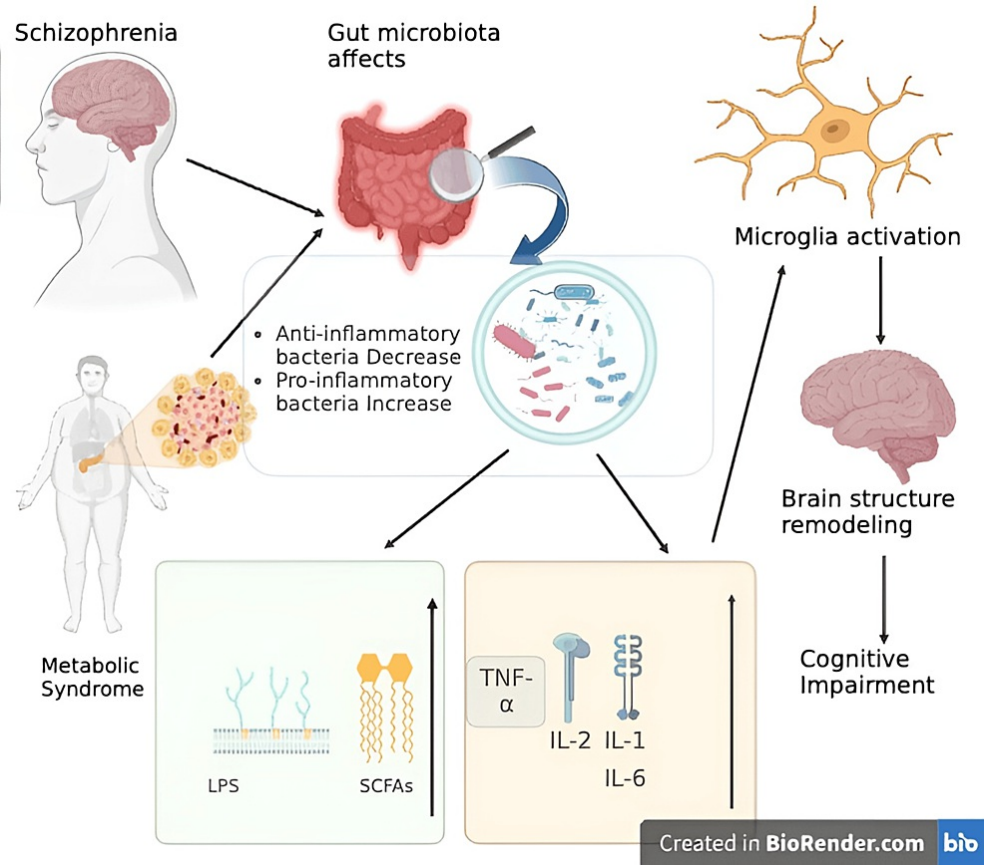
Pathophysiology of the link between metabolic syndrome and cognitive impairment in patients with schizophrenia LPS: lipopolysaccharide, SCFAs: short-chain fatty acids, TNF: tumor necrosis factor, IL-1: interleukin-1, IL-2: interleukin-2, IL-6: interleukin-6 Image credits: Ayushi Saxena Created in Biorender.com [12]

Figure 3 describes the pathophysiology of how metabolic syndrome in patients with schizophrenia may lead to an imbalance in gut microbiota, causing a decrease in anti-inflammatory bacteria, leading to an increase in interleukins and fatty acid chains, which might play a significant role in the microglia activation leading to the development of brain structure remodeling and ultimately cognitive impairment.

First and foremost, the gut microbiome plays a crucial role in maintaining a healthy metabolic and immunological function for the host. A disruption of the gut microbiota has been shown to contribute to obesity by influencing glucose/lipid tolerance and insulin resistance and promoting low-grade inflammation in the gut [18]. The pathophysiology of schizophrenia has been linked to similar processes [19].

Here is a brief explanation of the pathophysiology that links schizophrenia to cognitive impairment. Metabolic syndrome is highly prevalent in patients with schizophrenia, as discussed earlier in this article, and broadly impacts the gut microbiota. The large spanning effects of metabolic syndrome lead to a decrease in anti-inflammatory bacteria and an increase in the pro-inflammatory bacteria in the gut. These alterations in gut microbiology may further lead to responses like an increase in TNF-α, IL-1, IL-2, and IL-6 levels. This increased stress might also lead to increased lipopolysaccharides and short-chain fatty acids in the body. These changes further lead to microglial activation in the brain as these are the immune cells of the brain, which ultimately leads to brain structure remodeling and subsequently may lead to cognitive impairment [15,18,19]. Furthermore, dysbiosis of gut microbiota may result in the release of pro-inflammatory cytokines into the bloodstream, which may then cross over the blood-brain barrier in some cases, leading to neuroinflammation, including microglia proliferation. This neuroinflammation has been observed in both schizophrenia and obesity and is associated with changes in brain neurological functions [20].

Furthermore, imbalanced gut microbiota could activate the hypothalamic-pituitary-adrenal (HPA) axis, which may result in increased cortisol levels and decreased levels of brain-derived neurotrophic factor [21]. The HPA axis, part of the gut-brain axis, responds to stress by triggering a series of hormonal reactions, including corticotrophin-releasing hormone, adrenocorticotropic hormone, and cortisol. The autonomic and limbic systems regulate it and act in a feedback loop. Cortisol affects many biological systems and has a circadian cycle with the highest levels in the morning, known as the cortisol awakening response. Surprisingly, the growth of the gut microbiota coincides with the establishment of this circadian cycle. Dysregulation of the HPA axis is widespread in severe mental illness, most likely as a result of stress exposure. Changes in gut microbiota and increased intestinal permeability may be significant causes. Stress can cause dysbiosis in the gut, increased permeability, and activation of the HPA axis, contributing to inflammation. Interactions with gut microorganisms and autonomic ileal corticosterone production also play a role. However, the specific consequences of HPA axis activation on gut microbiota and permeability, as well as the relationship between gut microbiota alterations and a wide spectrum of stress-related metabolic dysregulations, remain unknown. The brain volume of patients with schizophrenia and obesity is also reduced as a result of both of these factors [5,20,21].

The findings indicate that an impaired metabolic state could worsen the neurocognitive deficits associated with schizophrenia, particularly in the domains of symbol coding and spatial span [10]. Previous research has shown varying cognitive impairments in schizophrenia patients with metabolic syndrome compared to those without metabolic syndrome [10]. According to Kern et al., schizophrenia patients exhibited less impairment in reasoning and problem-solving abilities but experienced significant deficits in processing speed and working memory [13].

**Figure 4:**
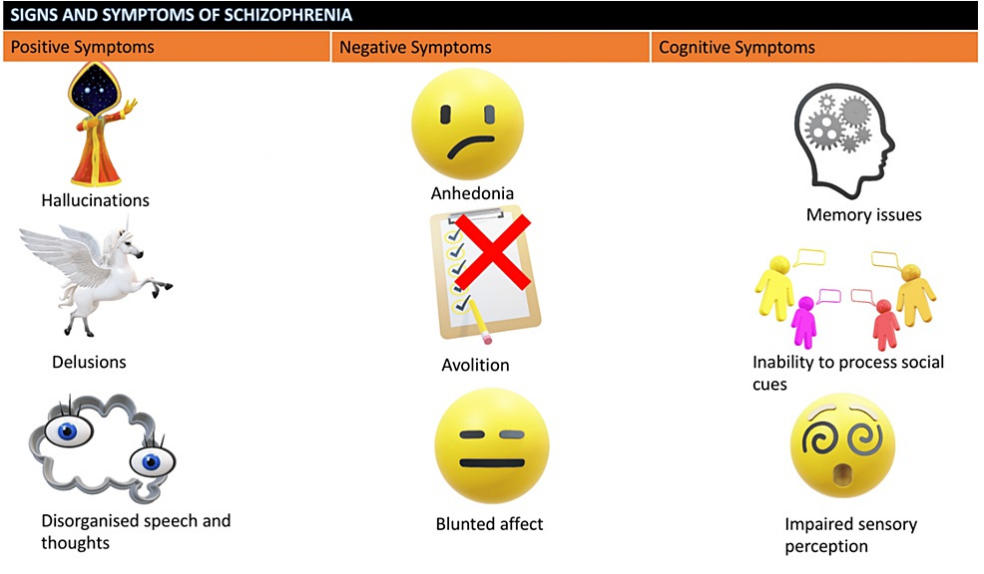
Schizophrenia is explained by its impact on a person Image credits: Ayushi Saxena Created in Powerpoint

In a study conducted by Ojeda et al., it was discovered that when information processing speed was considered, impaired working memory was no longer identified as a hallmark of schizophrenia [14]. In this study, a total of 100 people with schizophrenia and 53 healthy adults participated, which comprised neuropsychological tests encompassing six cognitive domains, including processing speed, attention, verbal memory, visual memory, working memory, and executive functioning [14]. The fit of a 6-factor cognitive model was evaluated using confirmatory factor analysis (CFA). CFA results confirmed the hypothesized 6-factor cognitive organization. Across all measures, there were cognitive differences between patients and controls, as expected. When the effects of processing speed were considered, the differences in the other five cognitive domains were significantly reduced. While correcting for other characteristics resulted in a much smaller reduction in group differences, even these effects were largely explained by variations in processing speed [14]. This shows that the major cognitive loss in schizophrenia is slowed processing speed. Furthermore, our findings suggest that metabolic syndrome may affect the symbol coding T score. Based on these findings, we cautiously speculate that metabolic syndrome may slightly affect information processing speed in individuals with schizophrenia [14]. Figure 4 illustrates the positive and negative symptoms of schizophrenia. It can have various cognitive symptoms as well.

Eyler et al. recently reported that cognitive therapy and management of metabolic syndrome might help delay cognitive decline among individuals with schizophrenia [22]. The frequency of metabolic syndrome in chronic schizophrenia patients is usually reported to be anywhere from two to four times greater than in the general population [22]. Furthermore, reversing metabolic syndrome in these patients is challenging, even after discontinuing the antipsychotic treatment [22].

The findings from this and previous studies provide evidence supporting the importance of early metabolic risk management in schizophrenia patients to alleviate fundamental cognitive deficiencies, particularly those linked to information processing. As a result, it is critical in clinical practice to periodically assess glycemic and lipid metabolic parameters during antipsychotic medication, give counseling for a healthy lifestyle, and intervene to prevent delay or prevent the progression of metabolic syndrome [22]. This proactive approach aims to prevent metabolic syndrome from exacerbating cognitive deficits in individuals with schizophrenia.

Another research separated individuals into three groups depending on the existence of metabolic syndrome diagnostic criteria [23]. Compared to schizophrenia patients with normal metabolic function, individuals with both metabolic syndrome and metabolic disorders had considerably poorer cognitive impairment in symbol coding [23]. Similarly, studies involving the general population have found that an increasing number of items fitting the metabolic syndrome criteria is related to lower cognitive function [23].

**Figure 5:**
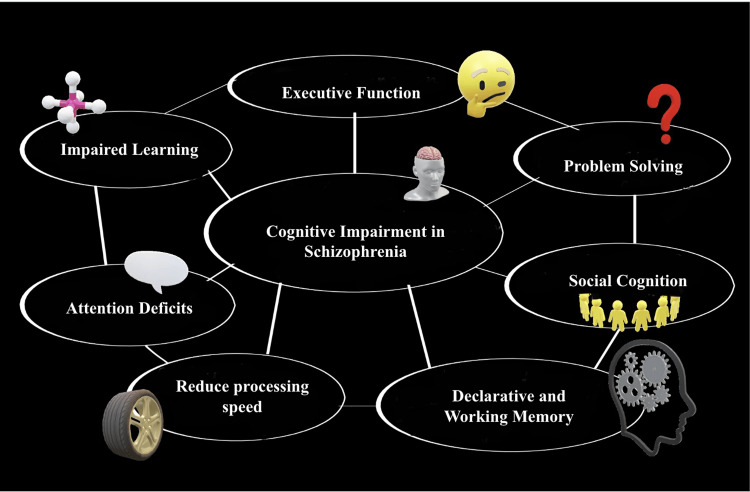
Cognitive impairment is a significant characteristic of schizophrenia, a complex and chronic mental disorder that affects a person's thinking, emotions, and behavior Image credits: Ayushi Saxena Created in Powerpoint

Simultaneously, having a history of three or more cardiovascular risk factors, such as obesity, hypertension, diabetes, and dyslipidemia, might increase the incidence of moderate cognitive impairment in older individuals by 1.58 times [24]. Furthermore, it doubles the probability of progression from moderate cognitive decline to dementia by 4.92 [24]. These studies give evidence that metabolic deficiencies have a cumulative effect on cognitive impairment. According to our further study, cognitive function tended to decline as the number of items meeting the metabolic syndrome criteria grew. These findings imply that metabolic syndrome may aggravate cognitive function in schizophrenia patients, notably in the symbol coding cognitive component [24]. Chen et al. explored the effect of several metabolic syndrome markers on cognitive function in chronic schizophrenia patients through a cross-sectional study. The study showed that neurocognitive performance was correlated with fasting plasma glucose, HDL cholesterol, systolic blood pressure, diastolic blood pressure, abdominal circumference, and triglycerides [25]. Notably, blood pressure, including both systolic and diastolic blood pressure, had effects on various cognitive domains, including speed of processing, working memory, problem-solving and reasoning abilities, verbal learning, visual learning, and overall scores on the MATRICS Consensus Cognitive Battery (MCCB). These findings align with previous reports that have linked elevated levels of fasting plasma glucose and blood pressure to cognitive impairments in processing speed, verbal learning, visual learning, and executive function in individuals with schizophrenia [26-28]. Figure 5 illustrates that schizophrenia is generally associated with a wide range of cognitive deficits, which can considerably impact daily functioning and quality of life for individuals with the condition.

In a study by Efimova et al., it has been observed that antihypertensive drugs have the potential to increase cerebral blood flow, leading to improvements in neurocognitive performance among individuals with metabolic syndrome from a study that included 54 patients with metabolic syndrome who underwent brain single-photon emission computed tomography, 24-hour blood pressure monitoring, and neuropsychological testing before and after 24 weeks of combination antihypertensive therapy, revealing that diffuse cerebral perfusion disturbances were associated with cognitive issues in metabolic syndrome patients, while antihypertensive treatment improved blood pressure, cerebral blood flow, and cognitive function [29]. According to a study by Green et al., results suggested that some aspects of social cognition were relatively intact in prodromal samples of patients with schizophrenia. According to the study, social cognitive impairment should appear early in the disease. As the disease develops, no additional damage or even possible improvement in this domain may be detected [30].

In a prospective study, first-episode schizophrenia patients were followed up for one year, and it was observed that their social cognitive performance remained relatively stable after the acute phase of the illness [31]. During the acute phase, patients with schizophrenia experienced significantly more severe social cognitive impairment than those in remission [32]. There are several potential reasons for these findings. Metabolic syndrome may primarily affect neurocognitive performance rather than social cognition. While the metabolic syndrome group exhibited significantly lower neurocognitive scores in areas such as processing speed, digital sequencing, attention span, memory, language acquisition, or visual comprehension, no significant differences were observed between the metabolic syndrome group and non-metabolic syndrome groups in terms of social cognition performance. One of the primary reasons could be that some of the individuals in the research experienced the metabolic syndrome for a shorter period of time or had already undergone partial medication treatments. These factors could have influenced the lack of widespread impact on cognitive performance, particularly in social cognition [33].

A study conducted by Kern et al. among individuals aged 33-55 years reported that participants with persistent metabolic syndrome exhibited worse cognitive performance over a 10-year follow-up period than those without metabolic syndrome [13]. However, no significant difference in cognitive performance was found between individuals with non-persistent metabolic syndrome and those who never had metabolic syndrome during the follow-up period [13]. It is important to note that the managing emotions domain of the Mayer-Salovey-Caruso Emotional Intelligence Test (MSCEIT) within the MCCB does not comprehensively assess social cognition in schizophrenia. Managing emotions is just one aspect of social cognition, including affect perception, social cue perception, theory of mind, empathy, and attribution style. Therefore, using a comprehensive tool for assessing social cognition is crucial in future research to gain a more complete understanding of social cognitive functioning in chronic schizophrenia patients with impaired metabolism [13].

Limitations

According to our systematic analysis, one constraint of most research is a very small sample size, which may reduce study efficiency but raise the danger of misleading negative findings. On the other hand, we cannot clearly differentiate the direction of correlations between cognitive function and metabolic syndrome in cross-sectional studies. Poor cognitive abilities could possibly be a risk factor for metabolic syndrome because of a lack of decision-making ability. Another key shortcoming in this study's design is that multiple comparisons were not corrected; therefore, we could not entirely rule out the possibility of the paper's stated false positive results. Therefore, to verify the results of this investigation, a prospective, longitudinal study with sufficient power is required in the future.

## Conclusions

In conclusion, mounting data point to a considerable connection between schizophrenia patients' cognitive impairment and metabolic syndrome. Dysbiosis of the gut microbiota, as a key component of the gut-brain axis, is implicated in both schizophrenia and metabolic syndrome, potentially serving as a crucial link between these two disorders. Obesity is a common issue among those with schizophrenia, and the two disorders have several potential correlations. Medication side effects, lifestyle variables, genetic predisposition, appetite regulation disorders, psychological factors, socioeconomic inequalities, hormonal changes, inadequate healthcare access, and the existence of co-occurring medical diseases are all included in this list. Antipsychotic medicines, particularly second-generation antipsychotics, have been linked to weight gain and metabolic abnormalities. Individuals with schizophrenia frequently have a poor diet, a sedentary lifestyle, and difficulty keeping a healthy routine. Genetic and psychological variables, including stress and emotional eating, may also play a role. Socioeconomic differences and hormonal shifts complicate the connection even further. Because obesity, the primary cause of a metabolic syndrome, offers considerable hurdles in the clinical management of schizophrenia, targeting the gut microbiota may present a unique therapeutic option for reducing obesity and improving mental outcomes in schizophrenia patients. However, further research is needed to fully elucidate the mechanisms underlying the intricate relationship between metabolic syndrome and schizophrenia. Various psychosocial and biological factors are involved, and the role of the gut microbiota as a therapeutic target requires careful exploration and consideration. Body mass index, smoking, drinking, exercise, food, and use of antibiotics or other medications with endocrine or immunomodulatory effects are a few examples of factors that need to be fully characterized together with the clinical characteristics of the patients. The effects of eating disorders, the influence of antipsychotic drugs on metabolic syndrome, and the existence of other chronic illnesses should all be taken into account. Furthermore, to guarantee the accuracy and comparability of the findings, larger research with defined procedures for sample collection and processing is required. Accurately identifying and profiling individual and disease-related traits associated with metabolic syndrome is crucial for understanding the intricate connections between the gastrointestinal microbiota and host health. The impact of metabolic syndrome on brain structure and function, as well as the longitudinal alterations in the intestinal microbiota of schizophrenia patients both prior to and following the onset of the metabolic syndrome, should be the primary areas of future research. Overall, this systematic review emphasizes the possible impact of metabolic syndrome and each of its separate elements on the neurocognitive functioning of schizophrenia patients. Metabolic syndrome is common in people with schizophrenia and tends to increase cognitive deficits, particularly those impacting processing speed and working memory. Emphasizing the importance of early management of metabolic risk factors emerges as a promising strategy to mitigate cognitive decline in this population. Monitoring metabolic parameters closely throughout antipsychotic medication, encouraging a healthy lifestyle, and proactively managing metabolic syndrome could all contribute to postponing or avoiding the emergence of cognitive problems. Furthermore, the cumulative influence of metabolic disorders such as obesity, hypertension, diabetes, and dyslipidemia, which can dramatically worsen different aspects of cognitive performance, must be recognized. Understanding these effects on many cognitive areas is critical for providing holistic treatment to those who have schizophrenia. Nonetheless, social cognition, a key component of cognitive abnormalities in schizophrenia, may be less directly impacted by metabolic syndrome. Future studies should dive deeper into this area, employing comprehensive evaluation techniques to acquire a more nuanced knowledge of social cognitive functioning in people with schizophrenia and metabolic issues. These results open the door to more focused treatment interventions to improve cognitive deficiencies in chronic schizophrenia patients. This study highlights the importance of early metabolic risk management in schizophrenia patients to alleviate fundamental cognitive deficiencies, particularly those linked to information processing. As a result, it is critical in clinical practice to periodically assess glycemic and lipid metabolic parameters during antipsychotic medication, give counseling for a healthy lifestyle, and intervene to prevent delay or prevent the progression of metabolic syndrome. This proactive approach aims to prevent metabolic syndrome from exacerbating cognitive deficits in individuals with schizophrenia.
